# Academic health sciences libraries and affiliated hospitals: a conversation about licensing electronic resources

**DOI:** 10.5195/jmla.2020.625

**Published:** 2020-04-01

**Authors:** Amy E. Allison, Bonita Bryan, Sandra G. Franklin, Leslie C. Schick

**Affiliations:** Associate Dean for Library Services, Geisinger Commonwealth School of Medicine, Scranton, PA, aallison@som.geisinger.edu, https://orcid.org/0000-0002-7552-1105; Head of Collection Services, Woodruff Health Sciences Center Library, Emory University, Atlanta, GA, libbrb@emory.edu; Director, Woodruff Health Sciences Center Library, Emory University, Atlanta, GA, libsrf@emory.edu; Senior Associate Dean and Director, Health Sciences Library, University of Cincinnati, Cincinnati, OH, leslie.schick@uc.edu, https://orcid.org/0000-0001-9352-2841

## Abstract

**Objective:**

Libraries in academic health centers may license electronic resources for their affiliated hospitals, as well as for their academic institutions. This study examined the current practices of member libraries of the Association of Academic Health Sciences Libraries (AAHSL) that provide affiliated hospitals with access to electronic information resources and described the challenges that the libraries experienced in providing access to the affiliated hospitals.

**Methods:**

In September 2016, AAHSL library directors received an email with a link to an online survey.

**Results:**

By December 2016, representatives from 60 AAHSL libraries responded. Two-thirds of the responding libraries supplied online information resources to more than 1 hospital, and 75% of these libraries provided the hospitals with access both on site and remotely. Most (69%) libraries licensed the same resource for both the academic institution and the hospitals. Cost, license negotiation, and communication with hospital stakeholders were commonly reported challenges.

**Conclusion:**

Academic health sciences libraries with affiliated hospitals continue to grapple with licensing and cost issues.

## INTRODUCTION

For students, faculty, and trainees in the clinical setting, the academic health sciences library provides online access to journals, books, and other information used for patient care and research. Many academic health sciences libraries also offer access to these online resources to the staff employed by the hospitals and/or clinics affiliated with the libraries’ institutions [1]. Experiences at the authors’ respective institutions show that making online resources available to these affiliated hospitals presents many challenges, such as accurately identifying the authorized user populations and financing the cost of licensing for these additional populations.

Access to online journals, databases, and books at affiliated hospitals has become an important and valuable resource for faculty, residents, and clinicians at academic medical centers. In a 2016 study by Quesenberry et al., faculty and residents at an academic institution reported using online resources when working on lectures, papers, and research [2]. In a 2011 survey administered at multiple hospitals, clinical staff identified electronic journals, PubMed, UpToDate, and electronic books—all online resources—as the most frequently consulted information resources used while providing patient care [3]. Access to online resources is also a highly valued incentive for preceptors who train students [4–6]. Many preceptors work at the hospitals affiliated with the academic institutions offering these clinical training programs. Likewise, the academic institutions’ faculty often practice at the affiliated hospitals. In 2007, Brown and Kaste surveyed academic health sciences libraries with an affiliated teaching hospital. Of the 55 responses received, 87% reported sharing licenses for some or all the library’s resources with the primary affiliated teaching hospital [7].

**Figure 1:**
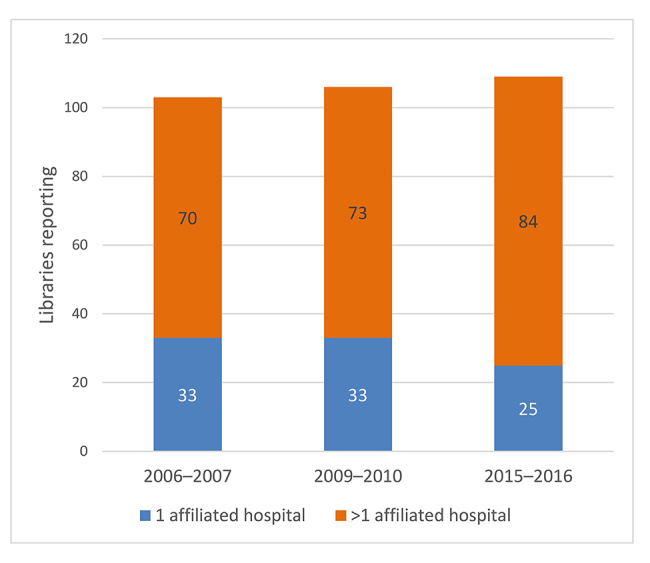
Number of teaching hospitals affiliated with an academic health sciences library’s institution Source: Association of Academic Health Sciences Libraries (AAHSL) Descriptive Statistics [1, 15, 16].

Hospital closings, acquisitions, and consolidations have resulted in expanding health systems and partnerships, and these expansions have included hospitals affiliated with academic health centers [8–13]. The American Hospital Association reported that from 2010 to 2015, the number of announced hospital mergers and acquisitions increased by 70% [14]. In parallel, the number of academic health sciences libraries reporting more than 1 affiliated hospital has increased over the past decade (Figure 1) [1, 15, 16].

The Association of Academic Health Sciences Libraries (AAHSL), the professional organization for academic health sciences libraries in the United States and Canada, periodically surveys member libraries. Annual surveys include questions about the libraries’ electronic collections, such as the number of serials and databases; the total costs for these resources; and the portion of the collection dollars spent on electronic resources. The survey also includes questions about the institutions’ affiliated hospitals [16]. However, these surveys do not elucidate how much of the library’s electronic collections are available to affiliated hospitals.

AAHSL also periodically administers two other instruments in tandem with the annual survey. The Services and Resources Survey Instrument asks the respondents to identify specific databases, library management systems, and federated search systems that the libraries license [17]. The question does not ask libraries to specify if products are licensed for staff at affiliated hospitals. The Descriptive Statistics Survey Instrument includes one question that specifically addresses electronic resources for affiliated hospitals.

The thirtieth edition of the AAHSL survey (2006–2007) asked whether the library provided databases, e-journals, and/or e-books (among other possible services) to affiliated teaching hospitals and whether the library charged the hospitals for these services. Of the sixty-seven libraries that responded to this question, forty-four provided databases, forty-three provided electronic journals, and forty-two provided electronic books [1]. This question was eliminated from succeeding editions of the survey.

In the thirty-third (2011) and thirty-sixth (2014) editions, the Descriptive Statistics Survey asked about library services that were provided to physicians and other staff at the hospital. One answer choice was that the library provided access to electronic resources but no other library services [15, 18]. The question in these two editions did not include an option to indicate that the library provided electronic resources plus other services. Therefore, it was difficult to draw a conclusion about the extent to which libraries were providing electronic resources to affiliated hospitals. None of the AAHSL instruments included questions about licensing practices or the scope of the library’s online collections that were made available to affiliated hospitals.

In 2007, Brown and Kaste administered a twenty-five-item questionnaire to AAHSL libraries to explore services and online resources that the academic health sciences libraries provide to their institutions’ primary affiliated teaching hospitals [7]. AAHSL defines primary affiliated teaching hospitals as those contractually bound to support clinical training of medical students and residents from the academic institution’s medical school [18]. The questions focusing on electronic resources addressed restrictions on where staff at the affiliated hospital could access the library’s online resources, who paid for the affiliated hospital to have access, whether the library and hospital shared the licenses, what publishers’ pricing models for hospitals were, and whether electronic resources were integrated into the hospital’s medical record.

Brown and Kaste’s survey responses represented 38% of AAHSL libraries at the time. In providing access to online resources, 60% of the respondents reported that the library paid the licensing costs, while 35% reported that the library and hospital shared the costs. At the majority (73%) of institutions, the library and hospital used the same authentication system to administer access, but no other information was provided about the technology used. According to Brown and Kaste, survey comments indicated that publishers’ tendencies to view affiliated teaching hospitals as distinct entities from academic institutions posed problems for libraries negotiating licenses. Other challenges included identifying whom to work with in the hospital on licensing and financing, as well as confusion about who had access to the resources.

Brown and Kaste’s study offered more details than the AAHSL surveys about how academic health sciences libraries licensed, funded, and administered access to electronic resources for affiliated teaching hospitals. However, Brown and Kaste only asked about a library’s practices related to its institution’s “primary affiliated teaching hospital,” whereas 77% of the 109 libraries with affiliated hospitals in the 2016 AAHSL Descriptive Statistics Survey reported having more than 1 affiliated hospital [7].

Finally, no previous surveys addressed how libraries dealt with nonfaculty providers associated with, but not employed by, the affiliated hospitals. Providers in private practice often admit and treat patients at affiliated hospitals. Because these private practice providers might not be part of the employee population at either the hospital or the academic institution, we wanted to explore practices concerning access to online resources for this group. This study aimed to build on the findings of previous surveys by describing current practices and challenges for academic health sciences libraries that license resources for multiple affiliated hospitals. Areas to be explored included:

- the scope of a library’s electronic collections licensed for affiliated hospitals,
- access for private practice providers,
- technology for administering access,
- challenges of licensing for affiliated hospitals, and
- practices that enable successful collaboration between academic health sciences libraries and affiliated hospitals.

## METHODS

After reviewing previous surveys, we developed an instrument with a combination of closed-ended and open-ended questions (supplemental appendix). We used a question format from AAHSL’s Descriptive Survey that allowed respondents to report the practices that they employed for each of their institutions’ affiliated hospitals [1]. We included questions about the technology used to administer access to the resources and how libraries handled access for private practice providers, defined in the survey as “health care providers who have admitting privileges but are not employed by the hospital.” Open-ended questions addressed the challenges of providing these resources to affiliated hospitals and factors that contributed to successful collaborations of libraries and affiliated hospitals. As a convenience sample, we used the AAHSL membership, which included libraries “serving accredited U.S. and Canadian medical schools belonging to or affiliated with the Association of American Medical Colleges (AAMC)” [19]. To encourage recipient participation, we limited the survey to eight questions.

A librarian and four academic health sciences library administrators who were not associated with the project previewed the survey and provided feedback on clarity and formatting of the questions. Prior to administering the survey, we sought and received an exemption from institutional review board (IRB) review. In September 2016, AAHSL library directors received an email with instructions and a link to the survey. We then emailed reminders during the following three months, and the survey closed in December 2016. Descriptive statistics were used to evaluate results from closed-ended questions. To analyze the open-ended questions, an investigator independently read the responses, identified themes, and classified responses according to the themes. A second investigator read the responses, reviewed the first investigator’s themes to confirm them or suggest different themes, and independently assigned one or more themes to each response. The two investigators compared their respective classifications and came to consensus on the themes assigned to each response.

## RESULTS

Of the 169 AAHSL member institutions surveyed in 2016, individuals from 60 (36%) libraries responded to the survey. Of the hospitals receiving access from survey respondents’ libraries, 89 were private, 56 public, and 1 was a children’s hospital. Only 7 of the libraries reported a mix of public and private hospitals.

**Figure 2:**
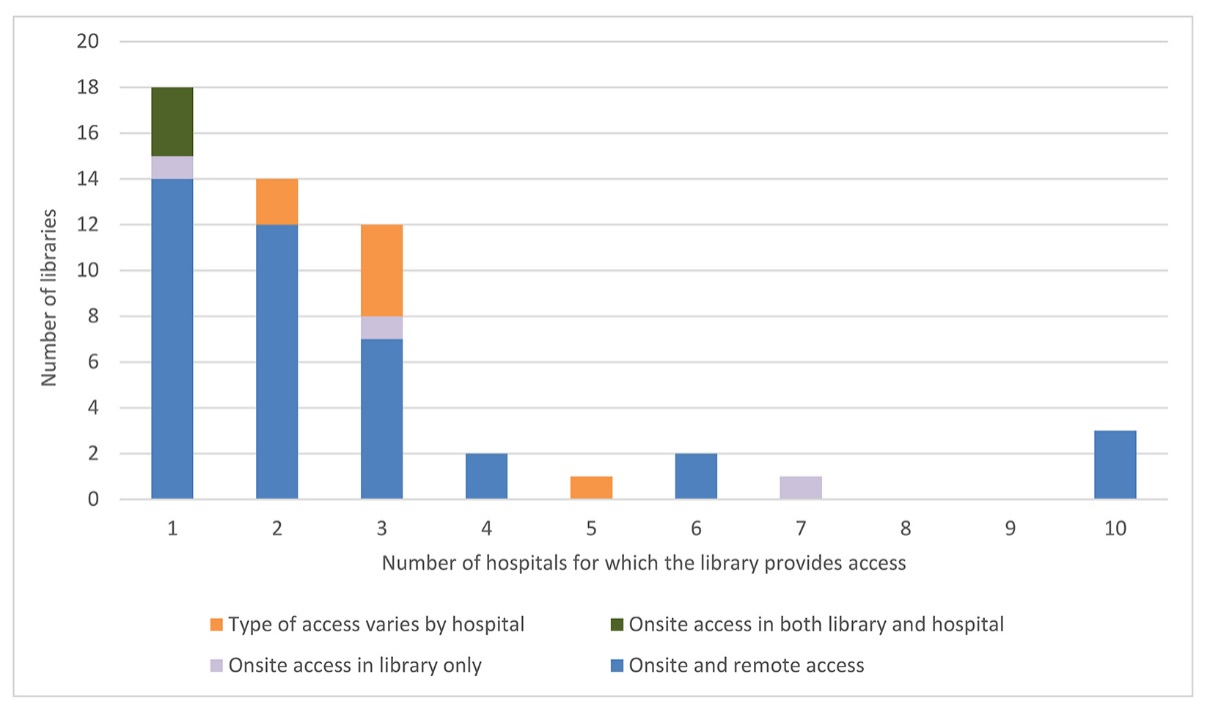
Libraries by number of affiliated hospitals and the type of online resource access provided to those hospitals

Of the 60 responses, 55 libraries licensed resources for a specific number of affiliated hospitals, 3 libraries did not license any resources for affiliated hospitals, 1 library was at an institution that did not have affiliated hospitals, and 1 response was unclear. Figure 2 depicts the distribution of the number of affiliated hospitals per library, as well as where hospital staff could access the resources: onsite or remotely, only when in the hospital and library, or only when in the library. Almost two-thirds (n=35) of the libraries supplied online information resources to more than 1 hospital. Most libraries (75%) provided access to these resources both on site and remotely, regardless of whether the library served 1 or multiple affiliated hospitals.

Of the fifty-one responses comparing the scope of resources licensed for the affiliated hospitals to the resources licensed for the respective academic institutions, thirty-five libraries reported licensing the same resources for both the academic institution and the hospitals (Table 1), but according to respondent comments, six of these thirty-five libraries made exceptions for some resources. Twelve libraries reported licensing a subset of the institution’s subscribed resources for the hospitals. Libraries serving one versus multiple hospitals had similar distributions of responses for this question.

**Table 1:** Summary of responses on scope of resources licensed, who negotiates licenses, and access provided to private practice providers

	All libraries reporting		Libraries with 1 affiliated hospital		Libraries with more than 1 affiliated hospital	
Scope of resources licensed for the hospitals	n=51		n=17		n=34	
All resources	35	(69%)	12	(71%)	23	(68%)
Subset of resources	12	(24%)	5	(29%)	7	(21%)
Varies by hospital	4	(8%)	0	(−)	4	(12%)
Who negotiates licenses	n=48		n=20		n=28	
Library assists the hospital in negotiating licenses for the hospital	2	(4%)	2	(10%)	0	(−)
Library negotiates shared licenses for the academic institution and hospital	41	(85%)	17	(85%)	24	(86%)
Library negotiates separate licenses for the academic institution and hospital	3	(6%)	1	(5%)	2	(7%)
Practice varies by hospital	2	(4%)	0	(−)	2	(7%)
Access for private practice providers	n=52		n=18		n=34	
No access	1	(2%)	1	(6%)	0	(−)
Access in the library only	7	(13%)	3	(17%)	4	(12%)
Access in the library and in the hospital	22	(42%)	8	(44%)	14	(41%)
Remote access	22	(42%)	6	(33%)	16	(47%)

Forty-eight libraries reported who negotiated licenses for library resources for their institution’s affiliated hospitals (Table 1). Most respondents (85%) answered that the library negotiated shared licenses for hospitals and the academic institution. When comparing libraries licensing for 1 hospital versus multiple hospitals, the percentage of libraries negotiating shared licenses was comparable (85% and 86%). Three (6%) libraries negotiated separate licenses for the academic institution and the hospitals. Only 2 (4%) libraries assisted in negotiating licenses for the hospitals, and each of these libraries had only 1 affiliated hospital. While only 2 (4%) respondents reported that the responsibilities for licensing varied by hospital, responses for 7 (17%) libraries negotiating shared licenses included a comment that the actual practice varied according to the product being licensed. Point-of-care resources and resources to be incorporated into the electronic health record system presented instances when typical licensing practices varied.

Regarding the type of access offered to private practice providers, 22 (42%) libraries provided remote access, and another 22 (42%) libraries provided access only in the hospital and library. However, among the 22 libraries providing remote access, 12 (55%) respondents commented that this access was only provided to private practice providers who were formally identified as fulfilling specific roles, such as preceptor. Seven (13%) libraries provided access only in the library.

Fifty-four libraries reported on the types of technology used for authentication and authorization (Table 2). The most common methods were proxy servers (49) and recognition by Internet protocol (IP) address (43). Regarding the number of different methods used for authentication, 30 (56%) libraries reported using 3 or more technologies.

**Table 2:** Authentication technologies used by libraries

Authentication technology	Number of libraries
Proxy server	49
Internet protocol (IP)	43
Virtual private network (VPN)	31
Shibboleth	12
Open Athens	2
Username and password for individual resource	1

Responses to open-ended questions provided insights into the challenges of providing access to online resources in the hospital environment (Table 3). Cost was the most frequently cited challenge in the survey (n=27), and respondents noted the high cost of clinical resources and the difficulty in obtaining funding from affiliated hospitals. Survey comments also described the difficulties in working with separate and differing information technology (IT) infrastructures and the challenges of negotiating shared licenses, such as hospital administrators’ unfamiliarity with licensing models for electronic information resources and the difficulty in defining authorized users from hospital system employee groups.

**Table 3:** Themes related to challenges in providing online resource access to affiliated hospitals

Theme	Subthemes	Sample respondent quotes
Cost (n=27)	High cost of clinical resources Hospitals unable, unwilling to pay Adding new hospitals, clinics Difficult to assign or distribute costs among the hospitals	“Everyone wants access, nobody wants to pay, and they act as if we obtain it for free.” “It is difficult to track usage from these sites since it is blended in with our campus users therefore challenging to assign costs to them.” “Growing costs and recent budget cuts.”
Working with hospitals (n=24)	Separate information technology (IT) units for the hospital and the academic institution Internet protocol (IP) ranges differ; difficult to keep track of changes to hospitals networks Security and firewall issues Separate authentication systems	“We have separate IT units, and they don’t communicate well with each other.” “There is some difficulty in keeping track of IP changes, firewalls, etc.” “Hospital does not have a good authentication system like EZproxy.”
Licensing (n=17)	Difficult to select subset of resources for hospitals Different license terms and cost models Difficult to define authorized users Need to educate hospital administrators about licensing library resources Language in licenses not addressing today’s health care organizations	“Sometimes it’s unclear whether a specific resource would be used at the hospitals or not.” “Publisher negotiations understanding that # of Beds and # of sites is not a reasonable methodology for pricing.” “publishers don’t understand what a health system is.” “Affiliate hospitals believe they have access to library resources after they affiliate.” “Educating our administration on license compliance.”
Communicating about access (n=10)	Difficult to obtain feedback from users about their needs Different methods for communication in the hospital and academic environments Confusion about available resources and how to access them	“receiving timely and constructive feedback on resources, access.” “Another challenge is communicating with hospitals users, as we are somewhat cutoff from a great number of them.” “With a mix of shared and institution specific resources, users are often confused about what they have access to and the correct route to access it.”

Survey respondents also commented on challenges related to communicating with hospital stakeholders about access (Table 3). They noted that academic institutions and affiliated hospitals often have different communication networks, so libraries may be “somewhat cutoff” from many of their hospital users. One respondent commented that “users are often confused about what they have access to and the correct route to access it.”

Participants identified practices (Table 4) that enabled academic health sciences libraries to work effectively with hospitals to provide access to hospital staff. These practices focused on communication and building relationships.

**Table 4:** Themes related to libraries’ practices enabling effective collaboration with affiliated hospitals

Factors contributing to effectively working with hospitals	Stakeholders and tasks
Build relationships with key stakeholders	Financial decision-makers Chief information officer (CIO)/chief medical informatics officer (CMIO) Hospital IT Staff who have influence with decision-makers
Understand the hospital environment	Technical environment, including restrictions on access, firewalls, and process for working with IT Organizational structure, including the nature of the hospitals’ affiliations with the university Process for budgeting and resource allocation User population: numbers and demographics of users, sites where users will have access, etc.
Engage with the user community	Discover needs and obtain feedback Improve awareness of resources and how to use them
Communicate strategically	Clarify to hospital stakeholders about terms of licenses, who will have access, what services and support the library will provide Document the level of financial support required from each hospital; include how costs are determined Educate decision-makers on how libraries operate and the nature of licenses for information resources Discuss with hospital IT the infrastructure required: IPs, authentication credentials, communication about changes to the network or systems impacting authentication and access Consider using a memorandum of understanding (MOU) to document in writing

Finally, 50 respondents answered a question about how the academic health sciences library marketed licensed electronic resources to hospital users. Twenty-five (50%) respondents reported that librarians marketed resources while they worked at the hospital or with specific user groups. Specific methods included hosting training sessions, working during resident reports or rounding, tabling at events, collaborating on research projects, or working on standing committees. Other frequently cited methods included newsletters (n=15, 30%) and hospital communication channels (n=14, 28%), such as intranets and bulletin boards or digital signs. Respondents also mentioned email (n=13, 26%) and the library website (n=11, 22%). Five (10%) respondents reported using social media, and 1 (2%) identified the electronic health record as a method. Seven (14%) respondents reported that the hospital libraries were responsible for marketing the resources.

## DISCUSSION

The purpose of this study was to describe and better understand the environment in which academic health sciences libraries provided online resources to staff at their institutions’ affiliated hospitals. As expected, most survey respondents reported providing electronic resources to more than one affiliated hospital. Among the libraries represented in the survey results, most provided both onsite and remote access to affiliated hospitals and licensed the same set of resources for the affiliated hospitals and the academic institutions, regardless of whether the libraries served one or multiple affiliated hospitals.

Neither Brown and Kaste’s survey [7] nor previous AAHSL surveys [1, 15–19] obtained information about how libraries provided electronic resources to private practice providers associated with the affiliated hospitals. In this survey, 84% of libraries reported providing some access to private practice providers, with 42% of libraries providing remote access. Some respondents indicated their institutions identified these providers as preceptors or volunteer faculty, resulting in the providers automatically receiving access to resources. In other cases, departments or schools must request that the library grant selected providers access to the library resources. One respondent noted that:

This is a tricky question. If the volunteer faculty or preceptor [has] a university network account, he/she may access our resources online and remotely. These groups are not automatically given accounts, the departments must request them.

These comments indicated the complexity in defining this segment of the health care population for licensing and providing access to electronic resources.

When asked how institutions authorized and authenticated users for access to online resources, most respondents reported that their libraries used a proxy server for managing access, along with several other methods. This finding represented the complex technology infrastructure that libraries often managed in order to connect authorized users with online resources when dealing with separate academic institution and hospital authentication systems. As observed in case studies, changes in IP ranges, navigation of security and firewall issues, or access to multiple identity management systems across the hospital and academic institution resulted in the library having to work with multiple IT departments or respond to rapidly changing IT infrastructure [20, 21]. Providing access in such a complex environment can test the library’s ability to maintain seamless access across the entire system and for all users.

In addition to the technology challenges involved in providing seamless access to electronic resources, survey respondents identified challenges related to funding licenses, negotiating licenses, and communicating about access issues. It was not surprising that cost was the most frequently cited challenge in the survey. During an initiative to consolidate electronic resources across multiple hospitals, Martin and Delawska-Eliott observed that extending the licenses for some electronic resources across the hospitals increased the costs over what the individual hospitals had previously paid [22]. The trend of hospital mergers and expanding health systems presents a budget challenge for academic health sciences libraries that are required to expand access to new sites and populations. Publishers often increase subscription costs when hospital populations are added to a system, and the financial impact can vary depending on the resource’s pricing model. One survey respondent described ongoing attempts to educate hospital system administrators that additional costs for information resources should be introduced during negotiations and be funded with the mergers. As an academic health sciences library’s user population increases, licensing all resources for the university and affiliated hospitals becomes more difficult to sustain.

Obtaining funding from affiliated hospitals can also pose a challenge. One respondent at a library with multiple affiliated hospitals reported that identifying the financial decision-makers in each affiliated hospital was difficult as was determining how to distribute costs to each affiliated hospital. A few comments specifically mentioned problems with collecting adequate usage data that could be correlated to specific hospitals. Standard vendor statistics, such as COUNTER, indicate what content a library’s authorized users are accessing and how frequently they access this content [23]. However, these statistics do not indicate whether the viewer of the content is a nonclinical faculty member, student, resident, or a pharmacist in the hospital. In our experiences, hospital administrators want data on utilization of electronic resources by hospital employees. Several respondents reported a perception that hospital administrators could not see the value of online resources or did not have funding available. As one respondent observed, many hospitals “have closed their libraries. They are in desperate need of resources and services, but do not have the money, or see it as overhead.”

Closely related to cost and budgeting was the challenge of license negotiations. Comments indicated that negotiating a single license for the academic and clinical units was difficult. It was not unusual for vendors to have different pricing and licensing models for clinical use. In our experiences, vendors might request total full-time equivalent (FTE) counts for specific user populations, such as medical students or residents; number of hospital beds; or numbers of in-patient admissions or out-patient visits. Vendors then developed a pricing model based on some combination of these and other variables. Respondents’ comments indicated it can be difficult to select resources to license for affiliated hospitals:

We have many system-wide joint licenses that are more academic rather than medical. Sometimes it’s unclear whether a specific resource would be used at the hospitals or not…We only license selected resources for the hospitals. It is difficult to track which hospital has access to what resource.

In addition to the providers and staff working in clinical departments, hospital staff include nurses, pharmacists, administrators, clergy, social workers, rehabilitation therapists, and others, each with their own specific needs for electronic journals, for example. Also, obtaining specific input from hospital staff and providers about what they need can be a challenge. Focus groups, surveys, and telephone interviews have been documented as methods for determining needs among hospital staff populations [22, 24]. Martin and Delawska-Elliott’s project [22] included the addition of 100 electronic journal titles based on work that included numerous stakeholder interviews and focus groups. Perley et al. used a self-reporting survey, interviews, and focus groups to determine needs across a multisite medical center population [24]. Data collection instruments allowed participants to report questions that they could not answer with available library resources or specific resources they could not locate. The extent to which the data identified specific titles or subjects for consideration was unclear in the report.

Some of the practices recommended by participants were similar to those that Brown and Kaste discussed [7]: establishing good relationships with stakeholders, educating administrators about license limitations, and developing formal agreements between the library and the hospital. Also, librarians can familiarize themselves with the hospital’s budget process and build relationships with the financial decision-makers to propose hospital financial support for online licenses. Appreciating how hospital administrators view funding, return on investment, and even strategic alignment can facilitate library negotiations with hospital administrators [25].

An understanding of the demographics of the hospital’s staff and the hospital’s infrastructure for administering access enables librarians to define authorized users and confirm for vendors how the libraries manage access to the resources. Librarians who negotiate joint licenses can help hospital administrators understand licensing terms and models and explain that licensing is not simply paying an additional per capita amount for every individual added to a license.

Respondents also noted that building relationships and maintaining communication with hospital IT can lead to more effective and efficient infrastructure solutions for supporting access. This supported Gentry and Marone’s finding that regular meetings of medical library staff and IT staff fostered solutions to technical issues and facilitated access to library electronic resources for the hospital staff [21]. Likewise, a survey of health sciences libraries, including academic and hospital libraries, found that respondents attributed successful collaborations with IT to “open communication,” an understanding of the expertise of staff in each department, and a “collegial environment” [26].

Ensuring that end-users were familiar with a library’s online resources and knew how to access those resources were persistent challenges observed by Martin and Delawska-Elliott [22] and Perley et al. [24]. O’Dell and Preston found that perceived barriers to access and unfamiliarity with resources stemmed from both insufficient communications from the library to the staff and assumptions by hospital staff about access [27]. Effective and continuous communication with end users can provide greater clarity about availability of resources and how to access them.

Also of note were recommendations by survey respondents to create a formal memorandum of understanding. Such a document can clarify mutually agreed upon expectations and responsibilities between the library and the hospitals, identify resources best suited for joint licensing, define the positions in the hospital and library with responsibilities for decision-making about licensed resources, and specify “a level of financial support” for licensed resources.

A limitation of this study was that the survey did not include an option for academic libraries that did not license resources for hospitals as well as an option for libraries at institutions that had more than ten affiliated hospitals. Indeed, several library administrators emailed the authors that they did not respond to the survey because their institutions did not license resources for hospitals. The response rate might have been higher if the survey had specifically included these answer options.

Due to the low response rate, the results of this survey might not completely represent the experiences of academic health sciences libraries. Also, although cost was the most frequently cited challenge, this survey did not investigate specific aspects of funding, such as how costs were shared. In the Brown and Kaste survey, when licenses were shared, 40% of the hospitals contributed to the licensing costs [7]. Finally, regarding an academic institution’s online resources, the survey did not differentiate between the health sciences library’s licenses and the university library’s licenses. Therefore, this survey did not capture information about how licenses that were shared among libraries at the academic institution might or might not be extended to the institution’s affiliated hospitals.

Licensing electronic resources for affiliated hospitals can be challenging for academic health sciences libraries. Further research is needed to determine how libraries fund online resources when asked to extend them to new hospitals. Other topics for investigation include:

- identifying data needed to make funding allocation decisions
- determining who in hospital organizations should be involved in making decisions
- defining the technology infrastructure needed to limit access by groups in the hospital and health system setting
- exploring impacts of licenses shared with other libraries in the academic institution on an academic health sciences library’s licensing for affiliated hospitals
- developing tools, such as templates for service agreements or memoranda of understanding, for facilitating communication with hospital administrators and other stakeholders

The goal of future work in this area should be to help academic health sciences libraries successfully adapt to changing health care environments and develop financially sustainable plans for providing information resources to academic institutions’ affiliated hospitals and health systems.

## SUPPLEMENTAL FILE

Appendix Survey: Licensing online resources for hospitals

## 

Amy E. Allison, AHIP*, aallison@som.geisinger.edu, https://orcid.org/0000-0002-7552-1105, Associate Dean for Library Services, Geisinger Commonwealth School of Medicine, Scranton, PA

**Bonita Bryan**, libbrb@emory.edu, Head of Collection Services, Woodruff Health Sciences Center Library, Emory University, Atlanta, GA

**Sandra G. Franklin, AHIP, FMLA**, libsrf@emory.edu, Director, Woodruff Health Sciences Center Library, Emory University, Atlanta, GA

**Leslie C. Schick**, leslie.schick@uc.edu, https://orcid.org/0000-0001-9352-2841, Senior Associate Dean and Director, Health Sciences Library, University of Cincinnati, Cincinnati, OH
