# Supplementary material for: Academic health sciences libraries and affiliated hospitals: a conversation about licensing electronic resources
Source: J Med Libr Assoc. 2020 Apr 1;108(2):242–52. doi: 10.5195/jmla.2020.625 (PMC7069815; doi:10.5195/jmla.2020.625)
Supplement: Appendix [file jmla-108-242-s001.pdf]

JMLA Journal of the Medical Library Association

SC

Supplemental content to
J Med Libr Assoc. Apr;108(2):dx.doi.org/10.5195/jmla.2020.625
www.jmla.mlanet.org
© Allison, Bryan, Franklin, Schick 2020

Academic health sciences libraries and affiliated hospitals: a conversation about licensing electronic resources

Amy E. Allison, AHIP; Bonita Bryan; Sandra G. Franklin, AHIP, FMLA; Leslie C. Schick

APPENDIX

Survey: Licensing online resources for hospitals

Instructions: The purpose of this survey is to collect information about academic health sciences libraries licensing electronic resources for hospitals. Your participation in this survey is voluntary, and your responses will not be linked to your institution. Aggregated results may be included in a paper. By submitting responses, you affirm that you grant consent to include your responses in this study.

The eight-question form requires ten to fifteen minutes to complete and is best answered by an individual with knowledge of the licensing practices of your library and general knowledge of what technology is used to authenticate and authorize users for access to electronic resources.

For the purposes of this survey, hospitals include any hospital for which the library participates in licensing resources, including teaching hospitals and community hospitals.

1. Consider the hospitals for which your library provides access to electronic resources. Please describe the type of hospital and how these hospitals receive access to library resources. *(Do not include separate hospital libraries to whom you do not provide resources.)*

For up to 10 hospitals, the answer choices for each hospital are:

Type of hospital:

- o Private
- o Public
- o Other

Access to online resources:

- o Online access in the library only
- o Online access in the library and hospital only
- o Access on site and remote access

2. Consider the hospitals you identified in question #1. Who negotiates licenses for library resources used by the hospital's community of users?

For up to 10 hospitals, the answer choices for each hospital are:

- o Your library negotiates licenses for academic institution and hospital; licenses are shared
- o Your library negotiates licenses for academic institution and hospital; licenses are separate
- o Your library assists the hospital to negotiate separate hospital use only licenses

Add comment.

JMLA Journal of the Medical Library Association

SC

Supplemental content to
J Med Libr Assoc. Apr;108(2):dx.doi.org/10.5195/jmla.2020.625
www.jmla.mlanet.org
© Allison, Bryan, Franklin, Schick 2020

3. If you license resources for the hospitals identified above, do you license the same resources as those you provide to your academic institution or a subset of these resources?

o All resources
o A subset of resources
o Varies by hospital

Add comment.

4. How do you authorize/authenticate users of your library's online resources? (check all that apply).

o OpenAthens
o Proxy server (EZProxy, etc.)
o Internet protocol (IP) recognition
o Each resource has a username and password
o Virtual private network (VPN)
o Shibboleth
o Other (Please specify)

Add comment.

5. Consider health care providers who have admitting privileges but are not employed by your hospital or academic institution (volunteer faculty, preceptors, etc.). Please describe how this group receives access to library resources?

o Online access in the library only
o Online access in the library and the hospital only
o Online access on site and remote access
o No access

Add comment.

6. What challenges do you face in providing online resources to the hospital user community?

7. In establishing a relationship with a hospital regarding access to electronic resources, what do you consider helpful and essential to the process?

8. How do you market online resources to your hospital's community of users?
